# Caring for people with dementia in rural Uganda: qualitative study of caregiving burden experienced by informal and formal caregivers

**DOI:** 10.29392/001c.12848

**Published:** 2020-06-16

**Authors:** Herbert E Ainamani, Paul E Alele, Godfrey Z Rukundo, Samuel Maling, Edith K Wakida, Celestino Obua, Alexander C Tsai

**Affiliations:** 1 Mbarara University of Science and Technology, Mbarara, Uganda; Bishop Stuart University, Mbarara, Uganda; Kabale University School of Medicine, Kabale, Uganda; 2 Mbarara University of Science and Technology, Mbarara, Uganda; 3 Mbarara University of Science and Technology, Mbarara, Uganda; Center for Global Health and Mongan Institute, Massachusetts General Hospital, Boston, Massachusetts, USA; Harvard Medical School, Boston, Massachusetts, USA

**Keywords:** uganda, sub-saharan africa, caregiving burden, alzheimer’s disease, dementia

## Abstract

**Background:**

The rising incidence of Alzheimer’s disease among older-age adults worldwide has been accompanied by an increase in caregiving burden. Limited work has examined the lived experiences of both formal and informal caregivers of people living with dementia in low-income countries.

**Methods:**

We conducted one-on-one, in-depth qualitative interviews with a purposive sample of 10 informal caregivers and 5 formal caregivers of people living with dementia in Mbarara, Uganda. They were interviewed about their experiences caring for people with dementia until thematic saturation was reached. All interviews were audio recorded, transcribed into English, and thematically analysed.

**Results:**

Two primary themes emerged from the data: patient factors influencing caregiving burden (problematic behaviours, such as wandering and aggression) and patient physical health and cognitive deterioration (namely, loss of memory and incontinence). Psychosocial and economic aspects of caregiving burden included financial costs, family conflicts, anxiety, stigma, and substance misuse.

**Conclusions:**

Both formal and informal caregivers of people living with dementia experience physical, financial, and psychological stressors. Interventions aimed at reducing these stressors would benefit caregivers as well as improve quality of care for people living with dementia.

Caring for people with Alzheimer’s disease and other related dementias (ADRD) demands considerable social economic efforts and adaptive behaviour from their care givers.^1^ According to Wimo et al, the annual global economic burden of caring for people with dementia was estimated to be at US$ 818 billion in 2015 and $1 trillion in 2018, with the highest burden prevailing in high income countries.^2^ Traditionally, the responsibility of caring for people with ADRD is borne by family members (eg, spouses or children), with fragmented involvement of formal caregivers.^3^ More than half of people living with Alzheimer’s dementia reside in low- and middle-income countries (LMICs).^4^ A recent study on dementia among older-age persons in rural Uganda estimated the prevalence to be 20%.^5^ ADRD exerts enormous negative consequences on the lives of caregivers and family friends, especially in LMICs where health systems are not well developed and under-resourced.^6–9^ Moreover, studies on dementia-related caregiving burden in LMICs report that caring for people with ADRD is correlated with increased caregivers’ stress, depression, and anxiety.^10,11^

While caregiving burden has been studied extensively in high-income countries, there is paucity of information about caregivers’ experiences in LMICs,^12–15^ particularly in rural areas of Africa where health systems for management of people with ADRD are under-resourced.^8,9,15–17^ Behavioural symptoms (e.g., agitation, wandering, and calling out repeatedly) are commonly exhibited by people with ADRD.^16^ In addition, caregivers whose patients are unable to perform activities of daily living may experience financial constraints, and both emotional and physical burden.^18–21^ Little research has explored the burden and lived experiences of both formal and informal caregivers of people living with ADRD in Africa. In this study, we define formal caregivers as healthcare providers (e.g., nurses, personal support workers, rehabilitation specialists, and physicians) who receive financial compensation to provide care,^22,23^ while informal caregivers include all those who provide caregiving unpaid (eg, relatives, friends, and family members).^24,25^ The aim of this study was to explore the burden of care and experiences of both formal and informal caregivers of people living with ADRD in rural Uganda.

## METHODS

### STUDY DESIGN

We conducted a qualitative study using a phenomenological approach, exploring caregivers’ lived experiences and using the data to understand the phenomena experienced.^26,27^ Similar to previously published phenomenological studies,^28,29^ we used a qualitative interview guide that included questions exploring the care burden experiences of both formal and informal caregivers. Two similarly-worded interview guides were used, with one designated for formal caregivers and one designated for informal caregivers. The interview guide for informal caregivers was translated into Runyankore-Rukiga, the local language of the region. The interview guide for formal caregivers was written in English because health care providers in the study setting communicate in English, the official national language.

### STUDY SETTING

The study was conducted at Reach One Touch One Ministries (ROTOM) Medical Centre in Rukiga District of southwestern Uganda. ROTOM is a “not-for-profit” non-governmental charity organisation with a medical centre that offers specialised care for older-age people. At the time of the study, the facility had about 350 older-age people under their care. ROTOM offers a variety of services, including provision of food/clothing and convening psycho-social groups that meet regularly at fellowship centres dedicated to ensure that older-age persons live with dignity.

### STUDY PARTICIPANTS AND RECRUITMENT

Using purposive sampling, we selected five formal caregivers and ten informal caregivers at ROTOM. Selection of formal caregivers was based on their roles and responsibilities, years of service in providing care for people with ADRD, and training; while informal caregivers were selected randomly using a list of caregivers provided by the ROTOM staff. ROTOM has “fellowship centres” where informal caregivers meet regularly. Only those who consented to participate were included in the study. Caregivers were interviewed until thematic saturation was achieved. Saturation was achieved more quickly in the sample of formal caregivers due to their more accurate reporting and efficient interviewing, as well as their structured working conditions.

To ensure comprehension and full awareness of the content, we collected oral and written informed consent from the participants. Participants who did not know how to read and write were permitted to indicate consent with a fingerprint. Each participant was interviewed in a private and quiet setting. Ethical approval for the study was given by the Mbarara University of Science and Technology Research Ethics Committee (07/10–18). Consistent with national guidelines, we obtained clearance to conduct the study from the Uganda National Council for Science and Technology (SS4893) and the Research Secretariat in the President’s Office.

### DATA COLLECTION

We collected data on the sociodemographic characteristics of study participants. Semi-structured interviews were guided by the interview guide (Online Supplementary Document). Formal caregivers were interviewed in English at ROTOM, while informal caregivers were interviewed in Runyankore-Rukiga at or near their homes. All interviews were audio recorded and supplemented with field notes. At the end of the interview, consistent with local etiquette and custom, and depending on the distance travelled and profession, caregivers received a study incentive in the amount of 20,000–80,000 Ugandan Shillings (approximately US$ 5–20 at the time of the study). The study was conducted between December 2018 and January 2019.

### DATA MANAGEMENT AND ANALYSIS

Data were transcribed verbatim by research assistants and compared with the audio recordings to verify fidelity of the transcription. The transcripts in Ruyankore-Rukiga were then translated into English by an independent translator. Any discrepancies in the translation were discussed and amended accordingly.

Two authors (HEA and GZR) independently read through the transcripts and developed codes and categories based on themes that emerged from the data. Initial coding was done by a single author (HEA), and then the codes were discussed with the other co-authors for consensus. A codebook was then developed and used to complete the coding. Two main themes emerged from our results: patient factors influencing caregiving burden, and psycho-social-economic aspects of caregiving burden. Correspondingly, we identified subthemes and categories, and selected quotations from both formal and informal caregivers to support our findings. Only three subthemes (cognitive deterioration, physical burden and financial-social burden) overlapped between the two categories of caregivers, while two subthemes (psychological and problematic behaviour) were identified and described by informal caregivers only. Discussions and conclusions in this study were based on subthemes, categories and quotations.

## RESULTS

A total of 15 in-depth interviews were conducted (5 with formal caregivers and 10 with informal caregivers). On average, formal caregivers had 5.6 years of caring for patients with ADRD while informal caregivers had 5.2 years of experience (Table 1). Codes, categories and themes generated from formal and informal caregivers yielded two major themes, and a number of subthemes (Table 2).

### THEME 1: PATIENT FACTORS INFLUENCING CAREGIVING BURDEN

The first dominant theme included a variety of factors that were associated with caregiving burden. Within this theme, two major subthemes included the patients’ problematic behaviours, and the patients’ physical health and cognitive deterioration. For ease of exposition, below and thereafter we refer to the subjects of caregiving as “patients” (with the understanding that they are formally defined as “patients” only for the formal caregivers).

### PROBLEMATIC BEHAVIOUR

None of the formal caregivers expressed concerns about their patients’ problematic behaviours. However, informal caregivers consistently described difficulties managing problematic behaviours (eg, confabulation, wandering and aggression), as described by one informal caregiver in reference to his mother:

*“My mother says things that aren’t true and it’s frustrating for us. She tells people how we don’t feed her, and steal her items including money. This kind of behaviour makes my family and I angry at her. We feel that we do our best for her and yet, we are not appreciated.”* (47-year-old man caring for his mother).

Informal caregivers also explained how caring for patients with dementia requires involvement from many family members so as to avert potential wandering behaviour. As described by one informal caregiver:

*“My father escaped from home. He wandered in places that we didn’t know. In the process, he was hit by a speeding motor bike and they called us to rescue him, which was very distressing for us as a family with apportioning blame for not taking care of him.”* (38-year-old man providing care for his father).

Informal caregivers voiced becoming upset when their patients became aggressive, either verbally or physically and in relation to themselves or other family members and friends. As an example, one caregiver had this to say:

*“You don’t know what to expect when a visitor comes to our house and tries to open up a conversation with my father-in-law. Many times, he tries to fight everyone, including family members. He’s very abusive and attacks everyone with all sorts of abusive words.”* (34-year-old woman providing care for her father-in-law).

Beyond verbally abusive behaviour, physically abusive behaviour was also described by many informal caregivers:

*“My mother was given a new mattress and pair of bedsheets by ROTOM staff. The following day, we found that she had sliced the mattress into pieces and the bedsheets as well. Her plan for the bedsheets was to make several pieces as tablecloths. When I asked her why she did that, she abusively reminded me that both the mattress and bedsheets were her property and that she had a right to use them the way she wanted.”* (63-year-old man providing care for his mother).

### PHYSICAL AND COGNITIVE DETERIORATION

Both formal and informal caregivers reported difficulty managing their patients’ physical and cognitive deterioration – namely, bowel/bladder incontinence, loss of memory, and physical immobility.

*“She wets herself and smears faeces and urine on the walls of the house. I have been forced to buy pampers and soap more often to clean her. It’s costly and not easy for me.”* (47-year-old man providing care for his mother).

*“Sometimes you have a patient in the diagnosis room or are administering treatment, and he/she asks for permission to go and ease him/her self, and it happens within a short time that the whole place is wet or soiled”* (37-year-old male formal caregiver with 12 years’ experience of providing care).

Informal caregivers described how their patients had lost memory, causing them to frequently wander and impose significant worry among family members:

*“My mother went out looking for her married children. She lost her way and spent a night in the banana plantation; this was tough for us as a family, because we spent the whole night searching for her.”* (52-year-old woman providing care for her mother).

Patients’ memory losses imposed different types of burden among formal caregivers. Formal caregivers expressed frustration with patients whose memory loss caused them to be nonadherent to medication or who could not provide reliable histories about presenting complaints. For example:

*“Our patients have problems with recalling and also understanding where it’s hurting most. They find it difficult to describe the pain and this affects our diagnosis more often, however, we have managed the patients with difficulty most of the time”* (29-year-old female formal caregiver with 4 years’ experience providing care).

Physical immobility also imposed significant burden on caregivers, because patients frequently needed support to move from one location to another. One informal caregiver described her experiences thusly:

*“My mother needs support to move from one place to another. One day I left her seated outside our house and went to pick a banana from our plantation. It started raining shortly after I had left. Knowing that she couldn’t move to the house, I rushed to rescue her. Unfortunately, it was too late. I found her very wet, and this was one of my saddest moments during my period of caring for her.”* (44-year-old woman providing care for her mother-in-law)

These mobility limitations were compounded when patients and families lacked wheelchairs. Formal caregivers did not experience similar day-to-day burdens but did encounter problems with physical exertion during examinations and treatment. For example, one formal caregiver noted:

*“When such patients come here, we participate in lifting them alongside the family caregivers. We also monitor the patients and advise the family caregivers to help in changing the patients’ positions since their body movements have been incapacitated. However, those who come without family caregivers, we do the work ourselves.”* (29-year-old female formal caregiver with 5 years’ experience providing care).

### THEME 2: PSYCHOSOCIAL AND ECONOMIC ASPECTS OF CAREGIVING BURDEN

Caregivers’ narratives also identified a variety of psychosocial and economic aspects of caregiving burden. We identified three subthemes: (1) physical burden, such as lower back pain and physical exhaustion; (2) financial and social burden, due to excessive costs (e.g., transportation, diapers and bedding), social isolation, and family conflict; and (3) psychological burden, resulting from stigma, hypervigilance and substance misuse.

### PHYSICAL BURDEN

Both formal and informal caregivers stated that caring for people with dementia affected their physical well-being. Caregivers described how manually lifting patients from one place to another, both in the hospital and at home, involved repetitive use (and potential strain) of the lower back. Formal caregivers generally engaged in these behaviours as a matter of professionalism. As described by one formal caregiver:

*“When they arrive here, we have to bathe them, carry them to the bathrooms, and sometimes it’s difficult to have them volunteer to take a shower. We have to carry them by force. This is physically straining and has affected my back.”* (29-year-old female formal caregiver with 5 years’ experience providing care)

Although formal caregivers described having pain related to lifting patients, informal caregivers generally described more severe difficulties in this regard, as they were involved in activities of daily living at home. One informal caregiver had this to say:

*“When I am not at home, he stays indoors all the time, because no one else can carry him. I can’t remember how many times I have carried him for the last 3 years. I feel physically tired and my back is hurting too. The doctor gave me medicine that I am taking now.”* (38-year-old man providing care for his father).

### FINANCIAL AND SOCIAL BURDEN

Both informal and formal caregivers described how caring for patients with dementia had negative financial and social implications. For example, informal caregivers incurred the costs of purchasing special foods, diapers for their patients, and paying to transport their patients to medical appointments. Formal caregivers frequently gave money to informal caregivers struggling with these costs and also experienced conflict with informal caregivers over caregiving roles. As described by one formal caregiver:

*“Patients come to us when they are hungry, and sometimes we have had to pick money out of our own pockets to feed them. Other times, family members call us, saying that patients are critically ill, we have to get to their homes by all means, and usually they don’t have reimbursement for our transport costs.”* (37-yearold male formal caregiver with 12 years’ experience providing care).

Informal caregivers found these costs to be very burdensome:

*“My father-in-law asks for special foods, like fish and meat, and yet I can’t afford the cost. I have to take care of my children’s school tuition as well. I have tried to borrow some money whenever he requests special meals, but most times I fail. My husband’s job is casual, and he does not have a salaried job, which leaves us with inconsistent income.”* (34-year-old woman providing care for her father-in-law).

Most informal caregivers described how their caregiving responsibilities required them to discontinue full-time employment; their social lives also became significantly circumscribed due to their need to remain confined at home for patient monitoring and caregiving. These restrictions are illustrated in the following quotation:

*“All I had been doing stopped. I am a trained electrician, but I have been compelled to stay at home because my mother can easily escape. I have also feared to get married because I am not sure if my wife will cope with my mother’s situation.”* (35-year-old man providing care for his mother).

Formal caregivers also noted that their roles as professional caregivers sometimes brought them into conflict with families over caregiving roles. Informal caregivers described having to spend time educating informal caregivers about successful aging and the importance of living in older age with dignity. Some formal caregivers even suggested that informal caregivers engaged in behaviour that could be perceived as potentially hastening the death of older-age family members:

*“I was supposed to pick the patient from his home for medical review and the family members soaked all his clothes in water so that he fails to get what to wear while going to the medical centre. This was done with the intention of denying the patient medical attention so that he dies so fast to save themselves from financial burden. This act was annoying. I had to reschedule the Doctors’ appointment and decided to embark on educating family caregivers about the importance of letting their elderly family members age gracefully.”* (28-yearold female formal caregiver with 4 years’ experience providing care).

In the same way, formal caregivers expressed their disappointment with informal caregivers whose actions led to the death of their patients:

*“We had a case where a patient was poisoned, and they announced his death. Fortunately, when we arrived at his home, he was still alive. Our intervention made the patient live for an extra four years. This was very annoying and the family members hurled insult on us. We have resolved to work hard in educating the community about taking care of people with Alzheimer’s disease.”* (37-year-old male formal caregiver with 12 years’ experience providing care).

On the other hand, both formal and informal caregivers described conflicts over roles and responsibilities of caregiving. For example, in most Ugandan families, the primary responsibility for caregiving is borne by adult children (eg, son or daughter-in-law) or adult grandchildren, and in some instances a single adult child will bear sole responsibility despite the capacity for other adult children to also share the responsibility:

*“We are five daughter in-laws, all the other four are free to engage in their private activities. I am confined here. While distributing property, we got the same share as the other ones doing nothing. When I ask them to help us sometimes and stay at home with grandma, they refuse and sometimes abuse me. This has more often caused conflicts between those families and mine.”* (44-year-old woman providing care for her mother-in-law).

Whereas informal caregivers experienced conflicts among themselves, formal caregivers experienced conflicts with informal caregivers who abandoned family members at the medical facility:

*“We have had to confront and conflict with family members of our patients over abandoning caregiving roles to us which interferes with our mutual relationships. They refer to their sick relative as ‘ours’, literally meaning that they belong to us. They assume that once we enrol patients into our program, we are solely responsible for the provision of the entire care. Yet our role is more technical. We expect them to provide the day-to-day care. However, they don’t do it, and this causes conflicts between us all.”* (37-year-old male formal caregiver with 12 years’ experience providing care).

### PSYCHOLOGICAL BURDEN

Informal caregivers described numerous experiences of being stigmatised by their communities and being uncomfortable with having friends come over for visitation because of their patients’ behaviour. Most informal caregivers described how their patients walked around the home naked or engaged in other embarrassing behaviours:

*“My mother walks around the neighbourhood while naked and everyone in the area says she’s ‘mad’, we have to keep monitoring her, she has turned a disgrace to our family since many people in the community are scornfully laughing at us.”* (52-year-old woman providing care for her mother).

Most informal caregivers described feeling anxious about their patients: they did not know what to expect in terms of erratic behaviours, they felt constantly on edge waiting for an abrupt call that their patients are have died (e.g. wandering about, being involved in a motor vehicle accident, or passing in their sleep):

*“There are times when I don’t sleep all night, thinking that my patient will die at any time as a result of committing suicide or even killing someone at home because he doesn’t differentiate between good and bad which makes me anxious and staying awake all the time”* (38-year-old man providing care for his father).

Furthermore, most informal caregivers expressed having difficulties in coping with the responsibilities of caregiving, including their patients’ incontinence. As a result, caregivers engaged in maladaptive coping behaviours, such as smoking and drinking:

*“I was not smoking and drinking before my mother became like this!! And as a way of forgetting my mother’s condition and gaining sleep, I drink and smoke to be able to gain some sleep and forget. I have found this helpful, for example: when I take my two glasses of Uganda waragi, I sleep till morning. I also find it fulfilling to be where others are, especially during drinking time.”* (63-year-old man providing care for his mother).

## DISCUSSION

In this qualitative study of formal and informal caregivers of patients with dementia in rural Uganda, our summative findings indicate that both informal and formal caregivers are highly burdened by their patients’ cognitive/physical deterioration (incontinence, loss of memory, physical immobility), leading to physical, financial and social burden (back pain, physical exhaustion, purchase of special foods/ diapers, social isolation, and conflicts over caregiving roles). Only informal caregivers appeared to experience caregiving burden related to their patients’ problematic behaviours (confabulation, aggression/destructive behaviour) and psychological sequelae such as stigma, anxiety/hypervigilance, and substance misuse.

Even though there were overlapping subthemes between both categories of caregivers, some of the subthemes were more prominent among formal vs. informal caregivers, and vice versa. For example, both formal and informal caregivers reported dealing with family conflicts. However, only formal caregivers reported encountering abandonment of their patients (by family members). In a systematic review that sought to explore how formal and informal caregivers can work together to strengthen the care triad, it was recommended that working with family caregivers required professionals to adapt to a different way of functioning.^30^ Further research is needed to provide specific recommendations for how formal and informal caregivers can work together to strengthen the care system and reduce family conflicts over caregiving.

Unlike formal caregivers who expressed frustration with patients who did not adhere to medication or otherwise engage in care, informal caregivers explained that problem behaviors led to a form of courtesy stigma^31^ and contributed to caregivers not wanting to have friends come over for a visit. This finding is consistent with one systematic review showing that cognitive decline contributes heavily to caregiving burden and general wellbeing.^1^ The psychological burden of this stigma is also consistent with previous studies that have found correlations between caregiving burden, stigma, depression, and anxiety.^10,32–35^ Moreover, informal caregivers in our sample explained that they were frequently overly alert, spending many sleepless nights fearing for unexpected patient disappearances or sudden death. This hypervigilance resulted in many caregivers’ inability to attend social functions thus leading to their relative social isolation.

Although our finding that informal caregivers engaged in substance misuse as means of coping with caregiving burden is consistent with previous studies,^36–38^ this finding can also be explained by the fact that social support of informal caregivers in our study was constrained by their relative confinement to their homes. Nevertheless, it remains important to note that the concepts of substance misuse, stigma, and anxiety were not discussed in detail within our study. Future quantitative studies should closely examine the association between caregiving burden and mental health concepts such as these within the context of rural communities in Africa.

### LIMITATIONS

Interpretation of our findings is subject to several limitations. First, the qualitative nature of our study design should be taken as hypothesis-generating rather than as hypothesis-testing. Second, certain biases common to retrospective designs may have affected the study participants, including recall and social desirability bias. Thirdly, our findings may not generalize beyond the specific study setting. Finally, although the families who receive services from this non-governmental organisation are largely similar to other families in the region, they most likely receive more support for family members with ADRD compared with families in more isolated rural areas. We recommend future comparison studies between caregivers who receive specialised formal/material support and those who do not.

## CONCLUSION

Caring for patients with Alzheimer’s disease and other related dementias is physically demanding, mentally draining, and financially burdensome to both formal and informal caregivers in rural Uganda. The formal caregivers in our study primarily described financial and physical burdens and conflicts with family members but not psychological burdens, whereas informal caregivers described the entire range of caregiving burden. Professional and informal support services, such as counselling, support groups, and public awareness campaigns may help to alleviate some of these challenges, along with appropriate financial support by all stakeholders.

## Supplementary Material

Ainamani_supplementary

## Figures and Tables

**Table 1.** Demographic characteristics of study participants

Sex	Age (years)	Formal education*	Relationship to the patient	Years of care
**Informal caregivers**				
Male	47	Primary	Mother	5
Female	44	Primary	Mother-in-law	7
Female	52	Primary	Mother	4
Male	38	Primary	Father	3
Female	64	None	Mother	6
Female	68	Teacher	Mother	4
Female	34	None	Father in-law	5
Male	35	Vocational	Mother	7
Male	63	None	Mother	6
Male	28	None	Grandmother	5
**Formal caregivers:**				
Male	37	Public health		12
Female	26	Nurse		4
Female	28	Clinician		4
Female	29	Nurse		5
Male	26	Physiotherapist		3

* All formal caregivers completed primary and secondary schooling; their occupations are listed in this column

**Table 2.** Themes, subthemes, and categories generated from the data

Theme	Subtheme	Categories
**Patient factors influencing caregiving burden**	Problematic behaviours	Confabulation
		Wandering
		Aggression/destructive behaviour
	Physical and cognitive deterioration	Incontinence
		Loss of memory
		Physical immobility
**Psychosocial and economic aspects of caregiving burden**	Physical burden	Lower back pain/physical exhaustion
	Financial and social burden	Transport funds to the hospital
		Diapers and bedding
		Special foods
		Social isolation
		Conflicts over caregiving roles
	Psychological burden	Stigma
		Anxiety and hypervigilance
		Substance misuse
