# Supplementary material for: Caring for people with dementia in rural Uganda: qualitative study of caregiving burden experienced by informal and formal caregivers
Source: J Glob Health Rep. Author manuscript; Available in PMC 2020 Oct 8. (PMC7544160; doi:10.29392/001c.12848)
Supplement: Ainamani_supplementary [file NIHMS1632101-supplement-Ainamani_supplementary.pdf]

ONLINE SUPPLEMENTARY DOCUMENT

Ainamani HE, Alele PE, Rukundo GZ, Maling S, Wakida EK, Obua C, Tsai AC. Caring for people with dementia in rural Uganda: qualitative study of caregiving burden experienced by informal and formal caregivers. J Global Health Rep, in press.

Interview guide

Dear Respondent,

My name is Herbert Elvis Ainamani, and I am a researcher from the Mbarara University of Science and Technology. I am going to ask you questions about your experiences providing care to people with Alzheimer's disease and other related dementias. You have been chosen to give your views and opinions on this topic. There are no right or wrong answers. We are only interested in your opinions. Your submissions will inform policy makers as well as health practitioners about the experience of caregiving burden.

Demographic data

1. [Record patient's sex]
2. What is your age?
3. What kind of formal education have you had?
4. What is you professional training?
5. [Informal caregivers only] How are you related to this individual with dementia to whom you are providing care?
6. How many years have you provided care for patients with dementia or your family member with dementia?

Interview prompts

7. Could you please share with us what it means to be a caregiver of someone with dementia?
8. Please tell us more about the kinds of changes you have noticed in your patient or relative since you first began providing care to him or her.
9. Could you tell us more about changes that have happened in your life as a result of providing care to such patients or to this family member with dementia?
10. Have you had any differences or conflicts with family members about caring for such patients with dementia, or have you had any differences with other family members in caring for your family member with dementia?
11. Is there anything else you want to say as far as taking care of these patients or family members is concerned?
